# Oral Administration of Heat-Killed Multi-Strain Probiotics Confers Durable Protection Against Antibiotic-Resistant Primary and Recurrent Urinary Tract Infections in a Murine Model

**DOI:** 10.3390/antibiotics14070634

**Published:** 2025-06-21

**Authors:** Bo-Yuan Chen, Zhen-Shu Liu, Yu-Syuan Lin, Hsiao Chin Lin, Po-Wen Chen

**Affiliations:** 1 Department of Veterinary Medicine, College of Veterinary Medicine, National Chung Hsing University, Taichung 40249, Taiwan; jackynike061548@gmail.com (B.-Y.C.); positiveshower@gmail.com (Y.-S.L.); shuan23274@gmail.com (H.C.L.); 2 Department of Safety, Health and Environmental Engineering, Ming Chi University of Technology, New Taipei City 24301, Taiwan; zsliu@mail.mcut.edu.tw; 3 Chronic Diseases and Health Promotion Research Center, Chang Gung University of Science and Technology, Chiayi 61363, Taiwan

**Keywords:** uropathogenic *Escherichia coli*, recurrent urinary tract infections, postbiotic, heat-killed probiotic, mice model, lactic acid bacteria, antimicrobial resistance

## Abstract

Background/Objectives**:** Alternative therapies for urinary tract infections (UTIs) have been explored, but their efficacy remains inconsistent. With rising antibiotic resistance, this study aimed to evaluate simplified postbiotic formulations derived from heat-killed probiotics for long-term protection against primary and recurrent UTIs in a murine model. Methods: We compared a multi-strain (seven-strain) versus a single-strain postbiotic in preventing *Escherichia coli*-induced UTIs and recurrent polymicrobial UTIs, assessed protection persistence after treatment discontinuation, and established a novel sustained UTI model via intravesical co-inoculation of three uropathogens. Mice were allocated to three experimental groups: a placebo group (PBS), Postbiotic I group (a seven-strain heat-killed probiotic formulation), and Postbiotic II group (a single-strain heat-killed probiotic). After two weeks of treatment, mice were challenged with uropathogenic *E. coli* (UPEC) and treated for seven days. Following a 14-day washout and bacterial clearance, they were rechallenged with multidrug-resistant UPEC, *Klebsiella pneumoniae*, and *Staphylococcus pseudintermedius*. Results: Both postbiotics significantly accelerated bacterial clearance in primary UTIs (*p* < 0.05). In recurrent UTIs, placebo-treated mice exhibited persistent bacteriuria, while Postbiotic I maintained a significantly higher sterile urine rate (50–80%, *p* < 0.01) post-treatment. Histopathological analysis confirmed reduced bladder and kidney inflammation (*p* < 0.05) with Postbiotic I. Conclusions: These findings demonstrate the superior efficacy of Postbiotic I in mitigating UTIs, with sustained protection post-treatment, supporting its potential as a long-term, non-antibiotic strategy. Additionally, our reproducible chronic UTI model, achieved through the co-inoculation of three uropathogens, provides a valuable tool for future research on chronic UTI pathogenesis and treatment.

## 1. Introduction

Urinary tract infections (UTIs) are among the most prevalent bacterial infections globally, affecting individuals across all age groups and genders in both community and healthcare settings [1]. For example, a recent study reported that the global prevalence of UTIs in older adults is 23.6% (95% Confidence Interval [CI]: 19.4–28.4). The highest prevalence was observed in specific subgroups, including individuals in Africa (30%; 95% CI: 12.7–55.8), women (30%; 95% CI: 14.6–51.7), those diagnosed via urine culture (25.3%; 95% CI: 18.3–33.8), and nursing home residents (47.2%; 95% CI: 24.2–71.5) [2]. Thus, UTIs represent a significant clinical concern worldwide.

UTIs typically begin when uropathogens colonize the urethra and ascend to the bladder, forming biofilms and evading immune defenses. Progression to the kidneys can also lead to bacteremia [3,4]. The most common uropathogens include *Escherichia coli*, *Klebsiella pneumoniae*, *Proteus mirabilis*, *Enterococcus faecalis*, and *Staphylococcus saprophyticus* [5]. Moreover, the standard treatment for UTIs involves oral antibiotics; however, prolonged use disrupts the vaginal and gastrointestinal microbiota and promotes multidrug resistance [6,7]. Moreover, although prophylactic antibiotics can reduce recurrent UTIs by up to 85%, concerns regarding antibiotic resistance and potential side effects still underscore the urgent need for alternative therapeutic approaches [8,9]. Importantly, distinguishing asymptomatic bacteriuria (ASB) from true UTI is crucial, as antibiotic treatment is often unnecessary for non-pregnant patients with ASB [4]. Collectively, non-antibiotic strategies—such as live probiotics, dietary supplements (e.g., cranberry, D-mannose), and herbal medicine—have shown promise, but their clinical efficacy remains inconsistent across studies, as further discussed below [10,11,12].

A recent randomized controlled trial found that probiotics, when used as an adjunct to conventional therapy in pregnant women with recurrent UTIs, significantly improved clinical indicators such as leukocyte and nitrite levels compared to placebo [13]. However, the small sample size (n = 60) limits the generalizability of these findings. A meta-analysis likewise found no conclusive evidence that probiotics outperform placebo or no treatment in UTI prevention [14]. While potential benefits cannot be entirely dismissed, the evidence remains limited and methodologically inconsistent. Comparisons with antibiotics also yield inconclusive results [14]. Although some studies report benefits in pediatric cases [15,16], a noninferiority trial in postmenopausal women showed that *Lactobacillus rhamnosus* GR-1 and *L. reuteri* RC-14 were less effective than trimethoprim–sulfamethoxazole, though they did not contribute to antibiotic resistance [17].

Overall, clinical evidence supporting the widespread use of probiotics for UTI prevention remains limited. Challenges such as selecting appropriate strains, determining optimal dosing, and ensuring product consistency continue to hinder broader clinical adoption [18]. These limitations highlight the need for alternative approaches. In this context, non-viable probiotic preparations (postbiotics) offer a promising strategy, with the potential to provide enhanced safety, stability, and immunomodulatory benefits—features that are explored in the present study.

Postbiotics, as defined by the International Scientific Association for Probiotics and Prebiotics (ISAPP), are “inanimate microorganisms and/or their components conferring health benefits” and offer several advantages over live probiotics, including enhanced safety and stability [12,13]. However, some reports continue to define postbiotics as “non-viable bacterial products or metabolic byproducts produced by probiotic microorganisms that exert biological activity in the host” [19]. To minimize ambiguity, in the present study, we define postbiotics as non-viable, non-replicating probiotic microorganisms, as only heat-killed probiotic cells were used. Nevertheless, the efficacy of postbiotics (inactivated probiotic cells) in preventing and treating tract UTIs remains largely unexplored. To address this gap, we first demonstrated that 10 heat-killed lactoferrin-expressing (LF-expressing) probiotic strains exhibited superior antibacterial efficacy compared to 12 naturally occurring probiotics. Furthermore, we showed that a three-week supplementation with a viable probiotic mixture (LAB), a heat-killed probiotic mixture (HK-LAB), or a heat-killed LF-expressing probiotic mixture (HK-LAB/LF) significantly reduced daily bacteriuria by 10^3^- to 10^4^-fold in a primary *Escherichia coli*-challenged UTI mouse model. These previously defined heat-killed probiotic formulations are referred to as postbiotics in the current study to ensure consistent terminology. Additionally, these treatments improved the bacteriological cure rate (BCR) with varying degrees of efficacy [20]. To further elucidate the therapeutic mechanism of postbiotic strains, we demonstrated that five additional postbiotic strains exhibited significant in vitro biofilm inhibition and dispersal activity. Notably, RT-qPCR analysis revealed that these inactivated probiotics downregulated genes associated with pili and biofilm formation (*fimA*, *csgA*) while upregulating genes involved in quorum sensing (*luxS*, *qseBC*, *sdiA*). These findings suggest that postbiotic treatment may inhibit pili and biofilm formation while promoting biofilm dispersion. Additionally, we have previously tested postbiotic composites containing 5 and 16 heat-killed probiotic strains. Both formulations significantly reduced bacterial load and morbidity in a primary UTI mouse model, with the 16-strain mixture reducing morbidity from bacteriuria by up to 30% within five days post-infection compared to placebo. However, preparing and producing formulations with higher numbers of strains involves greater complexity and labor intensity, which may hinder large-scale application. These findings provided the basis for the current study’s focus on a simplified postbiotic mixture containing either one or seven strains, selected for their potent anti-biofilm and antimicrobial activities, aiming to maintain comparable efficacy while enhancing production feasibility [15]. Furthermore, whether inactivated probiotics can effectively treat recurrent UTIs remains uncertain, particularly in cases involving severe antibiotic-resistant strains and high bacterial loads in urine.

In this study, we evaluate the efficacy of single-strain and a simplified seven-strain postbiotic formulation against both primary and recurrent UTIs. Based on our prior work with larger multi-strain heat-killed probiotic mixtures, we aim to determine whether a reduced number of selected strains can maintain comparable protective effects while improving practicality for production and application. Crucially, we also investigate the duration of protection conferred by these inactivated probiotics, assessing whether they provide sustained defense against recurrent UTIs even after a prolonged discontinuation period (two weeks). To our knowledge, this is the first study to demonstrate that distinct postbiotic formulations effectively prevent and manage both primary and recurrent severe antibiotic-resistant UTIs in a murine model, even after significant treatment cessation, highlighting their potential for long-lasting protection.

## 2. Results

### 2.1. Effect of Single- and Multi-Strain Postbiotic Formulations on the Improvement of Primary Urinary Tract Infections in Mice

**Figure 1:**
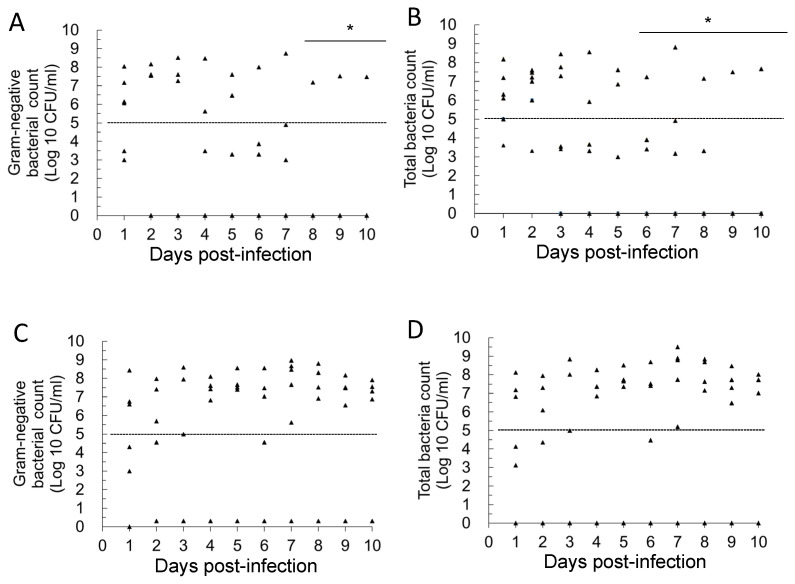
Daily changes in urinary bacterial counts in mice with primary and recurrent urinary tract infections in the placebo group. (**A**): Primary UTIs, total Gram-negative bacterial counts; (**B**): primary UTIs, total bacterial counts; (**C**): recurrent UTIs, total Gram-negative bacterial counts; (**D**): recurrent UTIs, total bacterial counts. * Mean bacterial counts were recognized to be significantly different from those on day 1 (*p* < 0.05). The dashed line in the figure represents a bacterial load threshold of 10^5^ CFU/mL. Each symbol in the figure (triangle) represents the urinary bacterial count from an individual mouse (n = 6 per group, sampled daily). In the log_10_ scale, values of 0 CFU/mL (i.e., no detectable bacteriuria) were arbitrarily assigned a value of 1 CFU/mL for graphical representation.

The gold standard for diagnosing UTIs is based on bacterial colony counts in urine [21,22]. Therefore, we assessed UTI severity in mice by measuring colony-forming units (CFU) in their urine. To monitor changes in bacterial load, we used both nutrient agar (NA) plates to determine the total bacterial count (TBC) and MacConkey (MCK) agar plates to quantify Gram-negative bacterial counts in daily urine samples. Our previous study confirmed that bacteriuria in healthy mice remains below 10^2^ CFU/mL [20], while a UTI is generally diagnosed when TBC in urine exceeds 10^3^ or 10^5^ CFU/mL. As shown in Figure 1, we analyzed the daily urinary bacterial counts in the placebo group for both primary and recurrent UTIs. In mice with primary *E. coli* UTIs (Figure 1A), statistical analysis revealed a significant reduction in urinary Gram-negative bacterial counts on days 8–10 post-infection compared to day 1 (*p* < 0.05). Similarly, TBC in urine was significantly lower on days 6–10 than on day 1 (*p* < 0.05). These results indicate a natural decline in bacterial load over time, with similar trends observed for both Gram-negative bacteria and TBC (Figure 1A,B). In contrast, in the recurrent UTI model, where mice were infected with a combination of UPEC, KP, and MRSP, a different pattern was observed (Figure 1C,D). Throughout the 10-day observation period, there was no statistically significant decline in daily urinary bacterial load (*p* > 0.05). Furthermore, compared to primary *E. coli* UTIs, most mice with recurrent infections exhibited persistently high bacteriuria, with urine bacterial loads often exceeding 10^5^ CFU/mL. These findings support the establishment of a sustained high-bacterial-load infection in the recurrent UTI model through mixed-pathogen bladder inoculation. To further characterize recurrent UTIs, we monitored bacterial loads for up to 14 days. However, for consistency in comparing primary and recurrent UTIs, we only present 10-day urinary bacterial counts in this figure. Nevertheless, persistently high bacterial loads were consistently observed in recurrent UTI mice throughout the full 14-day period.

**Figure 2:**
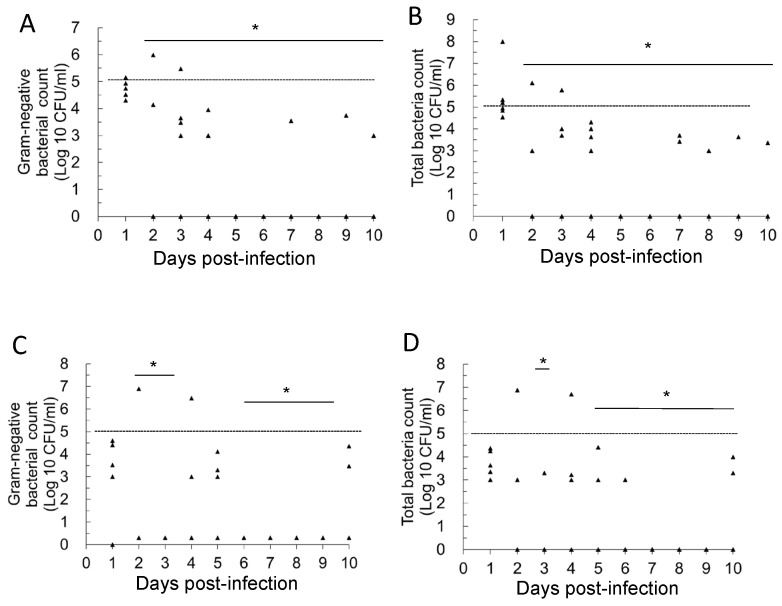
Daily changes in urinary bacterial counts in mice with primary and recurrent urinary tract infections in the Postbiotic I mice group. (**A**): Primary UTIs, total Gram-negative bacterial counts; (**B**): primary UTIs, total bacterial counts; (**C**): recurrent UTIs, total Gram-negative bacterial counts; (**D**): recurrent UTIs, total bacterial counts. * Mean bacterial counts were recognized to be significantly different from those on day 1 (*p* < 0.05). The dashed line in the figure represents a bacterial load threshold of 10^5^ CFU/mL. Each symbol in the figure (triangle) represents the urinary bacterial count from an individual mouse (n = 6 per group, sampled daily). In the log_10_ scale, values of 0 CFU/mL (i.e., no detectable bacteriuria) were arbitrarily assigned a value of 1 CFU/mL for graphical representation.

In the Postbiotic I group (Figure 2A,B; Primary UTIs), the statistical results show that from days 2 to 10 post-infection, the average number of Gram-negative bacteria and total viable bacteria in the daily urine of mice in this group was significantly lower compared to day 1 (infection establishment) (*p* < 0.05). In the secondary UTI model (Figure 2C), the average number of Gram-negative bacteria in daily urine was also significantly lower on days 2–3 and 6–10 compared to day 1 (infection establishment) (*p* < 0.05). Furthermore, in the same secondary UTI model (Figure 2D), the statistical results indicated that the total bacterial count (TBC) in the daily urine of mice in this group was significantly lower on days 3, 5–10 compared to day 1 (infection establishment) (*p* < 0.05). These results support the conclusion that the oral administration of Postbiotic I helps to reduce the bacterial load in bladder infections caused by primary *E. coli* infection. Importantly, even after discontinuing Postbiotic I for 14 days, the previous intervention continued to accelerate the reduction in bacterial load in the urine. In other words, oral administration of Postbiotic I not only has therapeutic effects on primary UTIs but also prevents secondary UTIs.

**Figure 3:**
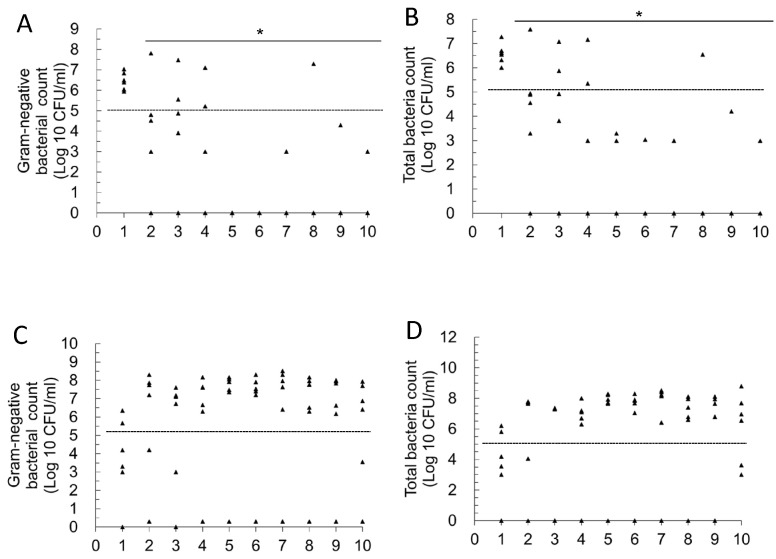
Daily changes in urinary bacterial counts in mice with primary and recurrent urinary tract infections in the Postbiotic II mice group. (**A**): Primary UTIs, total Gram-negative bacterial counts; (**B**): primary UTIs, total bacterial counts; (**C**): recurrent UTIs, total Gram-negative bacterial counts; (**D**): recurrent UTIs, total bacterial counts. * Mean bacterial counts were recognized to be significantly different from those on day 1 (*p* < 0.05). The dashed line in the figure represents a bacterial load threshold of 10^5^ CFU/mL. Each symbol in the figure (triangle) represents the urinary bacterial count from an individual mouse (n = 6 per group, sampled daily). In the log_10_ scale, values of 0 CFU/mL (i.e., no detectable bacteriuria) were arbitrarily assigned a value of 1 CFU/mL for graphical representation.

In the Postbiotic II group (Figure 3A,B; primary UTIs), the statistical results show that from days 2 to 10 post-infection, the average number of Gram-negative bacteria and total viable bacteria in the daily urine of mice in this group was significantly lower compared to day 1 (infection establishment) (*p* < 0.05). However, in the secondary UTI model (Figure 3C,D), statistical results showed that from days 2–10, the daily average of Gram-negative bacteria and total viable bacteria in the urine of mice did not differ significantly from day 1 (infection establishment).

### 2.2. Effect of Oral Administration of Two Postbiotics on Improving High Bacterial Load and Recurrent UTIs

**Figure 4:**
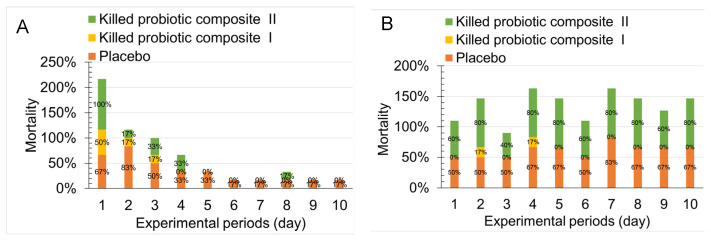
Differences in the proportion of mice developing significant bacteriuria (>10^5^ CFU/mL) following the induction of primary and recurrent urinary tract infections in the placebo, Postbiotic I, and Postbiotic II groups. (**A**): Primary UTIs, mice received either an oral placebo or one of the two postbiotics for 2 weeks prior to the induction of primary urinary tract infection, followed by continued postbiotic administration for 1 week. Bacterial load in urine was then evaluated 10 days after UPEC challenge. (**B**): Recurrent UTIs, on day 35 after the initial urinary tract infection (14 days after postbiotic discontinuation), mice were challenged again with a mixture of three uropathogens to induce recurrent infection with significant bacteriuria (>10^5^ CFU/mL). Bacterial load in urine was then evaluated 10 days after the challenge.

We analyzed the mean daily bacterial load between placebo, Postbiotic I, and Postbiotic II (Figure 2 and Figure 3) and aimed to assess the differences in the proportion of mice developing significant bacteriuria (>10^5^ CFU/mL) following the induction of primary and recurrent urinary tract infections (UTIs) across the treatments. As shown in Figure 4A, during primary infection, the placebo group showed a high bacteriuria proportion of 50–83% during the first 3 days post-infection. From day 4, the proportion decreased to 33%, and by day 6, it dropped further to 17%. However, by day 10, 17% of mice still exhibited high bacteriuria. In contrast, the Probiotic I group showed a high bacteriuria proportion of 17–50% during the first 3 days post-infection, but from day 4 onward, no mice in this group exhibited high bacteriuria (0%). The Probiotic II group showed a high bacteriuria proportion ranging from 17 to 100% during the first 3 days post-infection, but from days 5 to 7 and days 9 to 10, no mice in this group exhibited high bacteriuria (0%). These results demonstrate that after 2–3 weeks of oral administration of both postbiotic mixtures, the proportion of mice with high bacteriuria was significantly lower compared to the placebo group.

In Figure 4B, following bladder inoculation with three different pathogens to establish UTIs, the placebo group exhibited a high bacteriuria proportion ranging from 50 to 83% during the 10-day observation period, without any natural decline in this proportion. In contrast, the Probiotic I group only exhibited 17% high bacteriuria on days 2 and 4, with no high bacteriuria mice observed for up to 8 days (0%). However, the Probiotic II group showed a high bacteriuria proportion ranging from 40 to 80%, with infection rates similar to those of the placebo group. These results indicate that oral administration of Postbiotic I for 21 days followed by a 14-day discontinuation significantly reduced the occurrence of high bacteriuria in mice. On the other hand, while Postbiotic II reduced the incidence of high bacteriuria in primary UTIs, it did not reduce the occurrence of secondary UTIs caused by the three pathogens.

### 2.3. Histopathological Evaluation of the Selected Tissues in the Uropathogens-Induced Urinary Inflammation

**Figure 5:**
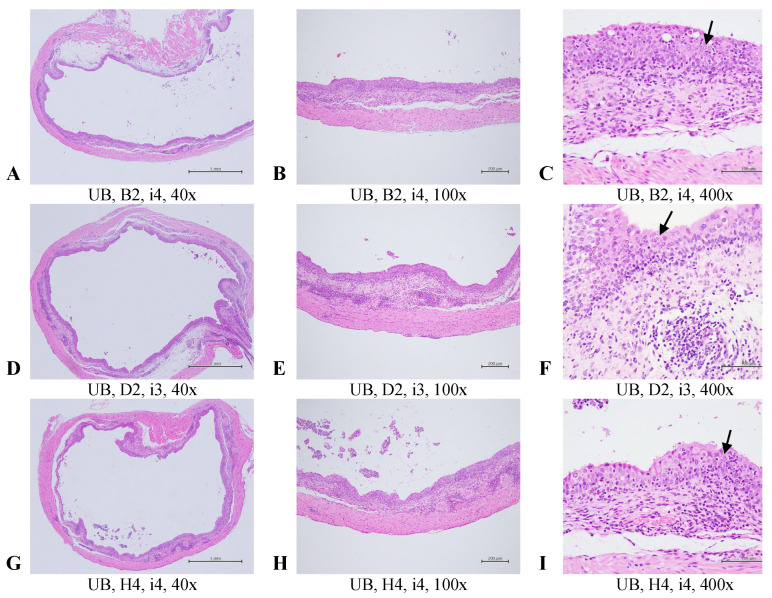
Representative images of histopathological examinations of the bladder in mice with recurrent UTIs treated with a placebo or one of two inactivated probiotic mixtures are shown. The groups are defined and explained in Figure 7. Bladder sections were stained with H&E, and images at 40×, 100×, and 400× magnifications are presented. Multifocal inflammation with mucosal hyperplasia in the urinary bladder was graded as moderate/severe (4) in the placebo group (**A**–**C**), moderate (3) in the oral Postbiotic I group (**D**–**F**), and moderate/severe (4) in the oral Postbiotic II group (**G**–**I**). Arrows indicate inflammatory cells.

**Figure 6:**
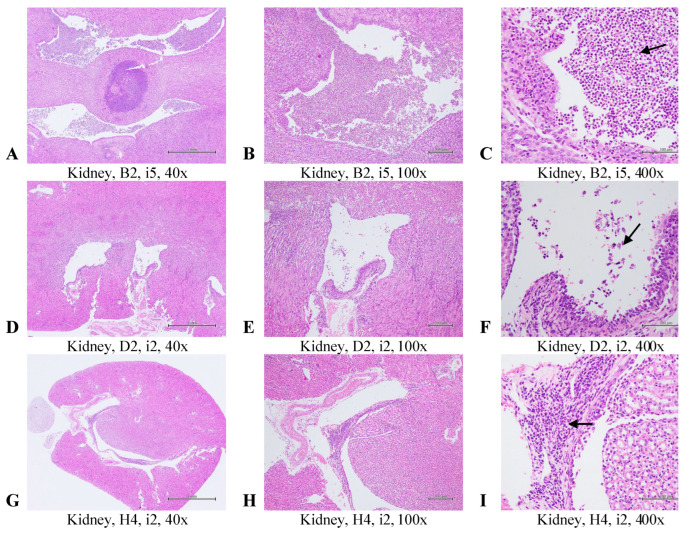
Representative images of histopathological examinations of the kidney in mice with recurrent UTIs treated with a placebo or one of two inactivated probiotic mixtures are shown. The groups are defined and explained in Figure 7. Kidney sections were stained with H&E, and images at 40×, 100×, and 400× magnifications are presented. Multifocal inflammation in the pelvis of the kidneys was graded severe/high (5) in the placebo group (**A**–**C**) and slight (2) in the oral Postbiotic I group (**D**–**F**) and oral Postbiotic II group (**G**–**I**). Arrows indicate inflammatory cells.

**Figure 7:**
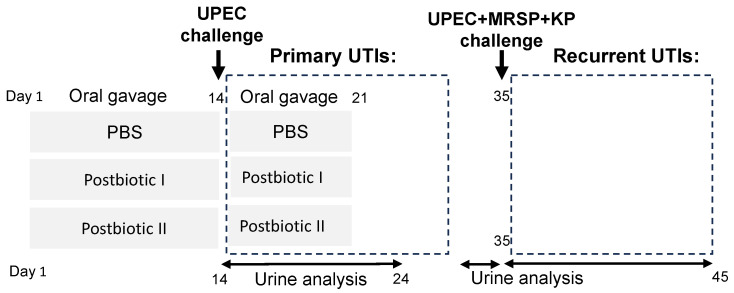
Experiment design. Mice were randomly assigned to three groups: placebo (PBS), heat-killed probiotic mixture I (Postbiotic I), and heat-killed probiotic mixture II (Postbiotic II) in oil. All groups received their respective treatments via oral gavage for two weeks. On day 14, mice were challenged with uropathogenic *Escherichia coli* (UPEC) via direct bladder inoculation. Treatment administration continued for an additional 7 days, followed by a 14-day washout period. Urine samples were collected daily for 10 days post-challenge (days 15–24) to assess bacterial burden. Two weeks later (day 35), mice were challenged with a polymicrobial mixture of uropathogens (*E. coli* UPEC, *Staphylococcus pseudintermedius* MRSP, and *Klebsiella pneumoniae* KP). Urine samples were collected daily for 10 days post-challenge (days 35–45) to quantify bacterial burden.

At the conclusion of the experiment, histopathological evaluations compared tissue changes among mouse groups after the reinfection course. Representative bladder and kidney tissue images are shown in Figure 5 and Figure 6. In the bladder (Figure 5), multifocal inflammation with mucosal hyperplasia was graded as moderate/severe (score = 4) in the placebo group, moderate (score = 3) in the oral Postbiotic I group, and moderate/severe (score = 4) in the oral Postbiotic II group. In the renal pelvis (Figure 7), inflammation scores were severe/high (score = 5) in the placebo group, slight (score = 2) in Postbiotic I, and moderate/severe (score = 4) in Postbiotic II.

Mean histopathological scores for bladder, kidney, ileum, and liver are then summarized in Table 1. Acute cystitis scores were significantly lower in the Postbiotic I group (0 ± 2.1) compared to the others (*p* < 0.05). Chronic cystitis scores (hyperplasia) were also significantly lower in Postbiotic I (0 ± 0) but did not reach significance (*p* < 0.05). Kidney inflammation scores were significantly lower (*p* < 0.05) in Postbiotic I (0 ± 0) and II (0.3 ± 0.8) compared to placebo (2.5 ± 2.1). Ileum samples showed no significant lesions, while liver evaluations revealed no lesions in the Postbiotic I and II groups and a slight lesion (score = 0.7 ± 1.6) in the placebo group. Overall, no significant differences were observed among the three groups in terms of ileum and liver histopathology.

These findings, consistent with bladder infection, demonstrate that Postbiotic I significantly reduces bladder and kidney inflammation while confirming the safety of both postbiotic formulations in the intestine and liver.

## 3. Discussion

This study aimed to evaluate whether a reduced number of selected postbiotic strains could maintain comparable protective efficacy while improving production feasibility, specifically in the prevention of primary and recurrent UTIs. We further investigated whether these formulations could confer long-lasting protection, particularly in the context of multidrug-resistant (MDR) uropathogens, and whether such effects would persist after a two-week discontinuation period. Our findings carry significant implications for the development of non-antibiotic interventions for UTIs.

A key finding of our study is that while the single-strain formulation (Postbiotic II, derived from *Lactobacillus gasseri*) exhibited protective efficacy against primary UTIs caused by *E. coli*, it failed to prevent recurrent infections induced by a polymicrobial challenge (UPEC, *Klebsiella pneumoniae*, and *Staphylococcus pseudintermedius*). Given that nearly 80% of clinical UTIs are attributed to *E. coli* infections [5], this suggests Postbiotic II may retain translational potential for simple infections. However, its limited effectiveness against recurrent or MDR-UTIs highlights the need for broader-spectrum strategies.

Importantly, our study design included a two-week washout period prior to reinfection, during which mice received no postbiotic treatment. This raises the possibility that continuous daily administration of Postbiotic II may be necessary to sustain protection against complex infections, a hypothesis warranting further investigation. In contrast, the multi-strain formulation (Postbiotic I), composed of seven probiotic strains, demonstrated consistent efficacy in both primary and recurrent UTI models. Remarkably, its protective effects persisted even after treatment cessation, suggesting that its mechanism of action may extend beyond direct antimicrobial activity and involve host immune modulation. Recent studies have shown that bacterial cell wall components in inactivated probiotics can activate dendritic cells and T cells, enhancing systemic immunity [23]. The diversity of cell wall structures in Postbiotic I may contribute to a more robust or sustained immunological response.

Beyond infection control, the role of postbiotics in modulating immune responses is gaining increasing attention. For example, in psychobiotic research, both live and heat-killed probiotics have been shown to improve emotional behaviors and reduce systemic inflammation in mice, even in the absence of overt pathology [24]. In our previous work, we demonstrated that sonication-killed *L. gasseri* HM1 (SK-HM1) and heat-killed HM1 (HK-HM1) significantly suppressed systemic IL-6, TGF-β1, and IL-1β expression, supporting their immunomodulatory potential. These earlier findings provide mechanistic support for the durable protective effects observed in the present study, even though cytokine levels were not directly measured here.

Although we did not assess systemic inflammatory markers such as serum cytokines in this study, UTIs are primarily localized infections, and systemic cytokine measurements may have limited explanatory power in this context. Recent studies have demonstrated that local urinary chemokines, rather than serum markers, better correlate with bacterial burden and tissue inflammation in murine UTI models, supporting the relevance of our chosen endpoints [25]. Instead, our histopathological evidence—showing reduced inflammation in the bladder and kidney tissues—provides a more direct and relevant indicator of therapeutic efficacy in the UTI model.

Our results also highlight the difficulty in treating MDR-UTIs. In the recurrent model, co-inoculation with UPEC, KP, and MRSP resulted in persistent bacteriuria and high bladder burden in placebo-treated mice. Antibiotic susceptibility testing confirmed that MRSP was resistant to all 22 antibiotics tested, while UPEC and KP were susceptible to only 6 and 4, respectively [26]. These data underscore the limitations of conventional antibiotics and support the use of adjunctive therapies. As Postbiotic I was administered orally and still achieved substantial bacterial clearance, it may serve as a candidate for co-administration with antibiotics to enhance treatment efficacy.

Histopathological analysis provided additional support for the protective effects of Postbiotic I. Mice treated with this formulation showed reduced inflammation in bladder and kidney tissues, consistent with microbiological findings. Notably, our previous in vitro study demonstrated strain-specific anti-biofilm activity among different heat-killed probiotics, supporting a multi-strain approach for targeting a broad spectrum of uropathogens [26].

The prolonged protection observed after Postbiotic I discontinuation challenges the prevailing notion that probiotic-derived therapies require continuous administration to remain effective. To our knowledge, this is the first report of a non-viable probiotic formulation retaining efficacy against MDR-UTIs beyond the active treatment period. Most alternative UTI interventions only confer protection during administration [10,11,12], making this an important advancement.

The safety of orally administered heat-inactivated probiotics has become an area of increasing interest, especially in the context of future clinical applications. Several clinical studies have demonstrated the safety and efficacy of heat-killed strains such as *Pediococcus acidilactici* K15 in reducing the incidence of respiratory tract infections in both preschool children and preterm infants without adverse effects [27,28]. In addition, comprehensive reviews have highlighted the reduced risk profile of heat-inactivated probiotics compared to their live counterparts, particularly in avoiding systemic infections, horizontal gene transfer, and interference with host microbiota [29]. Despite these advantages, it is important to recognize that probiotic effects and safety are strain-specific. Thus, further validation of the current postbiotic formulation in human studies is necessary. Furthermore, although most existing safety guidelines for probiotics—such as those proposed by Sanders et al. (2010) [30] and the ISAPP consensus statement [31]—primarily focus on live microorganisms, their core evaluation principles, including strain characterization, functional validation, and safety assessment, are also applicable to non-viable preparations. These frameworks will be valuable in guiding the design of future clinical trials assessing the safety and efficacy of heat-inactivated probiotics for UTI prevention.

We also established a simplified, robust chronic UTI model through transurethral co-inoculation of three uropathogens, which consistently induced high-bacterial-load recurrent infections in C57BL/6J mice. This method overcame the variability often observed in murine UTI models [32,33] and produced sustained bacteriuria in the placebo group, unlike the spontaneous resolution typically seen in monomicrobial infections. Previous attempts using dual-pathogen inoculation (UPEC + KP) failed to consistently induce recurrence [20], but the addition of MRSP proved critical. As a canine-derived uropathogen, MRSP may possess unique virulence traits that facilitate persistence. Future studies will assess MRSP’s independent pathogenicity and its role in chronic UTI pathogenesis.

Conventional treatment of uncomplicated UTIs primarily relies on oral antibiotics. However, prolonged antibiotic use can lead to long-term disruption of the vaginal and gastrointestinal microbiota and contribute to the emergence and spread of multidrug-resistant bacteria [6,7,34]. For example, several UTI-associated pathogens have been shown to develop high-level resistance to many first-line antibiotics [35,36], limiting both therapeutic and prophylactic options and increasing the overall cost of UTI management. Although low-dose, long-term antibiotic prophylaxis can reduce UTI recurrence by up to 85% [8], its clinical value remains debated. While such regimens may lower the risk of recurrent UTIs in children with prior infections, the benefit is considered modest and must be weighed against the risk of promoting antimicrobial resistance [9]. In light of these limitations, alternative strategies such as probiotics and postbiotics have gained increasing attention. Compared to existing treatments, live probiotics have been the focus of most clinical studies in the field of UTIs, as previously noted in the Introduction. However, their efficacy remains inconclusive; only a few studies suggest that live probiotics may help prevent UTIs, particularly in pediatric populations [15,16]. To our knowledge, no clinical trials have evaluated the efficacy of postbiotics (heat-killed probiotics) in humans for the prevention or treatment of UTIs. Our findings therefore provide critical preclinical evidence supporting future clinical investigations.

Studies using inactivated probiotics for UTI management have so far been limited to animal models. For instance, an early study by Asahara et al. demonstrated that the intraurethral administration of heat-killed *Lactobacillus casei* strain Shirota significantly reduced uropathogenic *E. coli* colonization following a single or multiple treatments. However, the same study reported no significant effect from several other *Lactobacillus* strains, highlighting strain-level variability in probiotic activity [37]. Importantly, the study by Asahara et al. did not evaluate the oral administration of inactivated probiotics, relying instead on intraurethral instillation to achieve local effects. While this route may provide direct antimicrobial action at the infection site, it is clinically less practical and more invasive than oral administration, especially for long-term prophylaxis or outpatient use. In contrast, our study demonstrates that the oral delivery of postbiotic formulations—composed of postbiotic strains—can confer significant protection against both primary and recurrent UTIs in a murine model. This finding has critical translational implications. Oral administration is more feasible and acceptable in real-world clinical settings, particularly for pediatric, elderly, or recurrent UTI patients. Moreover, our study further demonstrates that oral postbiotics remain effective even after treatment cessation, suggesting not only immediate therapeutic benefit but also prolonged host protection. These results position postbiotics as a promising non-antibiotic alternative for UTI management, potentially offering both efficacy and ease of use without the safety concerns associated with live probiotics.

Building upon our previous work, we further validated the potential of postbiotics in UTI treatment. In a prior murine UTI model, we showed that the oral delivery of heat-killed probiotic mixtures (HK-LAB and HK-LAB/LF) significantly reduced bacteriuria and improved cure rates, with better performance than live probiotics. Although ampicillin still yielded the highest cure rates, postbiotics—particularly when combined with recombinant lactoferrin—exhibited promising therapeutic efficacy and may serve as alternatives or adjuncts to antibiotic therapy [20]. Notably, these findings were obtained in the context of infections caused by a single *E. coli* strain, limiting their generalizability. The current study expands on these findings by demonstrating that simplified postbiotic mixtures retain comparable efficacy in a more complex and clinically relevant model of recurrent, polymicrobial, and multidrug-resistant UTIs. Importantly, postbiotic administration provided protection even after treatment cessation, underscoring its potential as a long-lasting, non-antibiotic strategy for recurrent UTI management.

In conclusion, Postbiotic I—a heat-killed, multi-strain probiotic formulation—provided superior protection against both primary and recurrent MDR-UTIs in mice. Its efficacy persisted post-treatment, suggesting potential immune-mediated mechanisms of action. Our novel polymicrobial UTI model further strengthens the translational relevance of these findings and offers a valuable platform for future research on postbiotic-based therapeutics.

## 4. Materials and Methods

### 4.1. Uropathogens

Three clinically relevant uropathogenic bacterial strains associated with UTIs were selected for this study. Uropathogenic *Escherichia coli* (UPEC; BCRC 10675), the most prevalent pathogen causing UTIs, and *Klebsiella pneumoniae* (KP; BCRC 10694) were obtained from the Bioresource Collection and Research Center (BCRC) in Taiwan. Additionally, a methicillin-resistant *Staphylococcus pseudintermedius* (MRSP) strain was isolated from a dog with a severe UTI. All bacterial strains were cultured aerobically at 37 °C for 16 to 18 h in Nutrient Broth (Difco™, BD, Franklin Lakes, USA). The antibiotic susceptibility of these three uropathogens was determined in our previous study [26], revealing that they are highly resistant to antibiotics.

### 4.2. Probiotic Strains

To evaluate whether a reduced number of selected postbiotic strains can maintain comparable protective efficacy while improving production feasibility, we developed two simplified inactivated postbiotic formulations based on our previous findings regarding the anti-biofilm activities of various probiotic strains against four key uropathogens [26]. In this study, Postbiotic I comprised seven inactivated probiotic strains, including *Lactobacillus delbrueckii* (BCRC 14008), *L. rhamnosus* (BCRC 16000), three isolates of *L. gasseri* (laboratory stock), *Ligilactobacillus salivarius* (laboratory stock), and *Lacticaseibacillus paracasei* (laboratory stock). In contrast, Postbiotic II was formulated by combining only the three *L. gasseri* isolates. The probiotic strains were first activated and anaerobically cultured in Lactobacilli MRS broth (Difco, Nutrient Broth, BD, USA) at 37 °C without agitation.

### 4.3. Preparation of Inactivated Probiotic Cells Through Heat Treatment

The preparation of inactivated probiotic and pathogenic cells followed procedures outlined in our previous report [20]. Briefly, selected probiotic strains were cultured and activated as described. Bacterial cells were then collected by centrifugation to yield approximately 5 × 10^10^ CFU, forming bacterial pellets. These pellets underwent two wash cycles with 25 mL of 1× PBS (phosphate-buffered saline with 0.1 M phosphate, 0.15 M NaCl, pH 7.2) using vortex homogenization for 15 to 30 s each. Subsequently, the suspension was centrifuged at 10,000× *g* for 10 min to remove the supernatant. The resulting bacterial pellets were resuspended in PBS and inactivated by autoclaving at 121 °C for 15 min. The Postbiotic I formulation was then prepared by combining seven inactivated probiotic strains in optimized proportions, while the Postbiotic II formulation consisted of three *Lactobacillus gasseri* isolates in varying ratios. The specific compositions have been refined based on internal research and are currently under patent application.

### 4.4. The Urinary Tract Infection Mouse Model

The schedule of the animal experiment is outlined in Figure 7. All animal experiments and protocols were conducted in accordance with the guidelines approved by the Institutional Animal Care and Use Committee at National Chung Hsing University (NCHU IACUC, approval numbers 111-014 and 111-105). Three-week-old female C57BL/6 mice were obtained from the National Laboratory Animal Center in Taipei, Taiwan. The mice were housed under controlled conditions at a temperature of 22 ± 2 °C with a 12 h light–dark cycle. They had ad libitum access to food and water and were acclimated to these conditions for four weeks prior to the start of the experiments.

To evaluate the efficacy of two postbiotic formulations in preventing primary and recurrent UTIs, a study was conducted involving 18 mice, randomly assigned to one of three groups: placebo (n = 6), Postbiotic I formulation (n = 6), and Postbiotic II formulation (n = 6). For 14 consecutive days (days 1–14, Figure 1), mice received daily oral gavage of either placebo (PBS), Postbiotic I formulation, or Postbiotic II formulation. Each daily dose consisted of approximately 1 × 10^10^ CFU-equivalent postbiotic cells in 200 μL PBS, administered via oral gavage. On day 14, all mice were challenged transurethrally with 1–2 × 10^8^ colony-forming units (CFU) of UPEC to induce primary UTIs, as previously described [20]. Post-challenge, mice continued receiving their respective treatments (PBS, Postbiotic I formulation, or Postbiotic II formulation) for an additional 7 days (days 14–21). Urine samples were collected daily for 10 days (days 14–24), starting one day after UPEC challenge. Fresh urine specimens were serially diluted tenfold (10^−1^ to 10^−6^), and aliquots were plated onto Nutrient Agar (NA) for total bacterial colony enumeration. Gram-negative bacterial counts were similarly determined using MacConkey (MCK) agar plates, with all plates incubated overnight at 37 °C.

On day 35 post-UPEC challenge, all mice were subjected to a second transurethral infection with a combined inoculum of 1–2 × 10^8^ CFU of UPEC, KP, and methicillin-resistant *Staphylococcus pseudintermedius* MRSP to establish recurrent UTIs. Urine samples were collected daily for an additional 10 days (days 35–45) and processed as described above to determine total bacterial and Gram-negative bacterial counts.

### 4.5. Histologic Analysis and Severity Scoring

Upon completion of the experiment, mice were euthanized, and selected tissues, including the bladder, kidney, ileum, and liver, were excised and fixed overnight in 10% neutral buffered formalin to evaluate the inflammatory status across different treatment groups. The fixed tissues were then embedded in paraffin, sectioned, and stained with hematoxylin and eosin (H&E). These stained sections were examined and scored by a blinded pathologist. The severity of inflammation was assessed using a grading scale from one to five. The severity of inflammation was assessed using a five-point grading scale, adapted from a previously published report [38]: 1 = minimal (<1% involvement); 2 = slight (1–25% involvement); 3 = moderate (26–50% involvement); 4 = moderate/severe (51–75% involvement); 5 = severe/high (76–100% involvement).

### 4.6. Statistical Analysis

To assess differences in bacterial load among urine samples, we performed a one-way analysis of variance (ANOVA), followed by an LSD post hoc test for pairwise comparisons to identify statistically significant differences between specific groups. Similarly, group differences in pathological scores were analyzed using ANOVA, followed by an LSD post hoc test for pairwise comparisons to determine significant variations between treatment groups. Statistical analyses were conducted using SPSS (version 20.0), with a significance threshold set at *p* < 0.05.

## Figures and Tables

**Table 1:** Histopathological assessment of tissue lesions following a recurrent UTIs model in mice. Mean ± SD is shown.

	Placebo ^1^	Postbiotic I ^1^	Postbiotic II ^1^	*p*-Value
Bladder -Inflammation, multifocal	2.3 ± 1.9 ^a^	0 ± 02.1 ^b^	3.7 ± 1.9 ^a^	0.003
Bladder -Hyperplasia, mucosa, diffuse	2.3 ± 2.1 ^a^	0 ± 0 ^b^	2.3 ± 1.2 ^a^	0.014
Kidney -Inflammation, pelvis, multifocal	2.5 ± 2.1 ^a^	0 ± 0 ^b^	0.3 ± 0.8 ^b^	0.008
Ileum	0.3 ± 0.5 ^a^	0 ± 0 ^a^	0 ± 0 ^a^	0.116
Liver -Inflammation, multifocal	0.3 ± 0.8 ^a^	0 ± 0 ^a^	0 ± 0 ^a^	0.391
Spleen -Hyperplasia, lymphocyte, multifocal	0.7 ± 1.6 ^a^	0 ± 0 ^a^	0 ± 0 ^a^	0.391

^1^ A recurrent UTI model was established as depicted in Figure 7, and following the experiment, all mice (n = 6 per group) were sacrificed. Histopathological lesions in selected tissues were graded based on severity: 0 = no abnormalities; 1 = minimal (<1%); 2 = slight (1–25%); 3 = moderate (26–50%); 4 = moderate/severe (51–75%); 5 = severe/high (76–100%). ^a,b^ Means with different letters in the same row are significantly different (*p* < 0.05) according to one-way ANOVA LSD test. Exact *p*-values are shown in the rightmost column.

## Data Availability

The datasets generated during and/or analyzed during the current study are available from the corresponding author on reasonable request.
